# Wearable Temperature Sensors Based on Reduced Graphene Oxide Films

**DOI:** 10.3390/ma16175952

**Published:** 2023-08-30

**Authors:** Xinyue Li, Tianrui Cui, Xin Li, Houfang Liu, Ding Li, Jinming Jian, Zhen Li, Yi Yang, Tianling Ren

**Affiliations:** 1 School of Integrated Circuit, Tsinghua University, Beijing 100084, China; xinyue9426@163.com (X.L.); ctr19@mails.tsinghua.edu.cn (T.C.); lixin919321@tsinghua.edu.cn (X.L.); lidingli0813@163.com (D.L.); jianjm@mail.tsinghua.edu.cn (J.J.); jacklee@tsinghua.edu.cn (Z.L.); 2 Beijing National Research Center for Information Science and Technology (BNRist), Tsinghua University, Beijing 100084, China; houfangliu@tsinghua.edu.cn; 3 Center for Flexible Electronics Technology, Tsinghua University, Beijing 100084, China

**Keywords:** temperature sensing, wearable electronics, reduced graphene oxide

## Abstract

With the development of medical technology and increasing demands of healthcare monitoring, wearable temperature sensors have gained widespread attention because of their portability, flexibility, and capability of conducting real-time and continuous signal detection. To achieve excellent thermal sensitivity, high linearity, and a fast response time, the materials of sensors should be chosen carefully. Thus, reduced graphene oxide (rGO) has become one of the most popular materials for temperature sensors due to its exceptional thermal conductivity and sensitive resistance changes in response to different temperatures. Moreover, by using the corresponding preparation methods, rGO can be easily combined with various substrates, which has led to it being extensively applied in the wearable field. This paper reviews the state-of-the-art advances in wearable temperature sensors based on rGO films and summarizes their sensing mechanisms, structure designs, functional material additions, manufacturing processes, and performances. Finally, the possible challenges and prospects of rGO-based wearable temperature sensors are briefly discussed.

## 1. Introduction

Body temperature is an important physiological signal. The metabolic process and internal environment stability of the human body are greatly influenced by temperature [1]. Low body temperature usually causes a decrease in vital signs [2], while high body temperature probably means fever and infection. Therefore, body temperature serves as an indicator for identifying abnormal bodily conditions. Measuring temperature is an effective method to assist in clinical diagnosis, and with the development of medical standards and social needs, has been playing important roles in health monitoring, disease prevention, and prosthetic skin [1,3]. In these applications, temperature sensors need to offer not only accuracy and precision but also flexibility and real-time monitoring capabilities. Hence, wearable temperature sensors, which combine all these properties, are regarded as a favorable option.

To create a consistent and comfortable interface between a wearable temperature sensor and body skin, the structure of the sensor should be carefully considered. In terms of dimension, sensor structures can be divided into 1D, 2D, and 3D forms, which are usually shown as wire [4,5,6], film [7,8,9], and bulk material [10,11,12]. Among them, 2D films are known for their large area and conformal contact compared with the other two forms, which leads to the higher reliability and accuracy of detected signals [13]. Flexible sensors based on films usually consist of a sensing layer, substrate layer (e.g., PI, PET, paper, or textile) [14,15,16,17] and encapsulation layer (e.g., PDMS, PI, or parylene) [18,19,20]. The sensing layer is deposited on the substrate layer, while the encapsulation layer acts as a protective layer to protect the whole sensor from environmental interference or damage.

As one of the determining factors of temperature sensing performance, the material of the sensing layer is cautiously selected. There are many wearable temperature sensors based on different sensing materials that have been reported, including metal-based materials [21,22], carbon-based materials [23,24,25], and conductive polymers [26]. Among them, reduced graphene oxide (rGO) is one of the typical temperature sensing materials in the carbon family. As a reduction derivative of GO [27], rGO has a great conductivity of ~7.2 × 10^3^ S/m [13] and an excellent thermal conductivity of ~5300 Wm^−1^k^−1^ [28]. It has a sensitive thermal response to resistance changes and an excellent temperature coefficient of resistance, thus making it an ideal candidate for temperature sensing. rGO is usually achieved by reducing graphene oxide (GO) through three main approaches: thermal, chemical, and optical reduction. Since there are always oxygen-containing functional groups remaining after the reduction process, measuring the degree of reduction is essential. X-ray photoelectron spectroscopy (XPS), Raman spectroscopy, and X-ray diffraction (XRD) serve as valuable techniques for characterizing rGO, offering information such as the O/C ratio and crystal structure. Due to the diversity of reduction approaches, many assembly methods, including coating, printing, electrochemical deposition, and infiltration, have been reported, which will be discussed in detail in Section 2.3.

As a temperature-sensitive material, rGO can be employed either independently or blended with other temperature-sensitive materials like metals, carbon-based materials, and conductive polymers to achieve enhanced sensing performance in temperature sensors. Besides their conventional role in body temperature sensing, wearable temperature sensors constructed using rGO films can expand their functionality to realize respiratory rate monitoring, finger touch detection, and body movement perception. This review discusses recent developments in wearable temperature sensors based on rGO films, including their manufacturing strategy, sensing mechanisms, additions of functional materials, and different applications. First, the synthesis, properties, and assembly methods of rGO are briefly introduced. Second, the performance of wearable temperature sensors based on pure rGO and mixed materials is discussed. Subsequently, some typical and advanced applications of flexible rGO temperature sensors are demonstrated. Lastly, a brief exploration of the potential challenges and future prospects associated with wearable temperature sensors based on rGO is provided.

## 2. Synthesis, Properties, and Assembly of rGO

### 2.1. Synthesis

**Figure 1:**
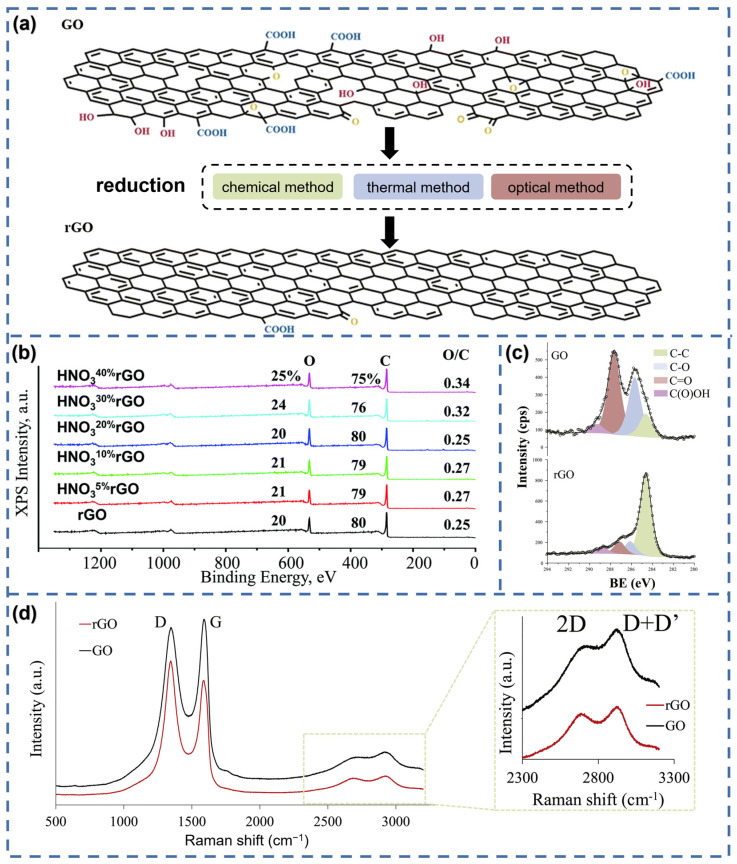
(**a**) Schematic of three main reduction methods of GO. Reproduced with permission from Ref. [34]. Copyright 2020 Institute of Chemistry, Slovak Academy of Sciences. (**b**) XPS spectra of the initial and HNO_3_-treated rGO materials. Reproduced with permission from Ref. [46]. Copyright Royal Society of Chemistry. (**c**) C1s region of the XPS spectra of the GO and rGO samples. Reproduced with permission from Ref. [44]. Copyright 2017 Elsevier B.V. (**d**) Raman spectra of GO and rGO including the D, G, 2D and D + D’ bands. Reproduced with permission from Ref. [44]. Copyright 2017 Elsevier B.V.

Epoxy, hydroxyl, carboxyl, and carbonyl groups are some typical oxygen-containing functional groups in GO (Figure 1a). Reducing GO is the normal way to produce rGO, while thermal, chemical, and optical methods are the three main reduction approaches [29,30]. rGO prepared via different methods shows differences in its physical, chemical, and electrical properties. Thermal reduction is usually carried out in vacuum, in an inert atmosphere, or with the presence of a reducing agent at a certain temperature for several hours. The defects in structure and the extent of the reduction are affected by the reduction temperature [31]. The thermal reduction method is limited in sensors that are based on textiles, since textiles usually cannot bear high temperatures. Thermal reduction at high temperatures results in a high C/O ratio and does not lead to impurities [32]. For example, Tu et al. measured the O/C ratio of GO films as a function of reduction temperature through XPS. It was observed that as the temperature was raised to 1000 °C, the O/C ratio decreased from 0.31 to 0.04 [33], indicating that higher temperatures result in a greater degree of reduction. Chemical reduction uses chemical reactions to remove the oxygenated functional groups in GO, which is the most traditional way to produce rGO. It can be achieved at room temperature or by moderate heating, providing an economical and convenient method for producing stable dispersions of rGO sheets [34]. Some of the most commonly used chemicals are hydrazine monohydrate (N_2_H_4_·H_2_O), ascorbic acid, sodium borohydride (NaBH_4_), and hydroiodic acid (HI) [35,36,37,38]. The highest C/O ratio reported of rGO prepared using chemical reduction methods does not exceed 15 at its maximum [31]. However, chemical reduction yields relatively higher electrical conductivity compared to thermal reduction [39]. Optical reduction uses lasers, ultraviolet light (UV), or plasma to raise the temperature and trigger the deoxidation reaction [31,40,41]. Plasma or laser irradiation has also demonstrated superior patterning capabilities for rGO compared to chemical reduction and thermal reduction [34]. By controlling laser power, scanning speed, line spacing, and other lasing parameters, a sustainable and scalable rGO pattern can be easily transferred onto any suitable substrate without destroying it [42]. 

It is important to mention here that functional groups in GO are impossible to remove completely through any methods. Thus, in order to obtain sensors with the best performance, it is meaningful to measure the degree of reduction through XPS, Raman spectroscopy, and XRD patterns to adjust and improve the reduction process. XPS spectra can be used to detect the ratio of elements contained. As for rGO, the O/C ratio is a key parameter that indicates the degree of reduction (Figure 1b). A lower O/C value means more thorough reduction due to the drop in oxygen content in the reduction process. Additionally, XPS spectra can demonstrate the peaks of different functional groups such as hydroxyls (C-O) and carbonyls (C=O). As is shown in Figure 1c, the contribution of non-oxygenated carbons (C-C) in rGO increases and becomes dominant in the C1s signals compared with GO, which indicates efficient reduction. Raman spectroscopy is another way to characterize rGO. The G-band (at ~1580 cm^−1^) and D-band (at ~1350 cm^−1^) are two characteristic bands in the Raman spectra of graphene and its derivative. The G-band is caused by the in-plane vibrations of sp^2^ carbon atoms, while the D-band is induced by the defects or disorders in the graphene lattice [43]. The D-band shift and the invariance of the G-band are typical of defective graphene materials [44]. The ratio of D-band and G-band intensities (I_D_/I_G_) depends on the defect density. In Figure 1d, the I_D_/I_G_ of GO and rGO are 0.96 and 1.14, respectively. The increase in I_D_/I_G_ indicates the transition from GO to rGO. Last but not least, XRD patterns also provide useful information about rGO. In the process of converting from GO to rGO, there are two typical peaks in XRD patterns (usually near 11° and 25°) that correspond to the (002) reflection of graphite and its derivatives [45]. The diffraction peak angles determine the interlayer distance according to Bragg’s law. A higher intensity of the peak near 11° indicates the presence of more oxide groups, while the higher intensity of the peak near 25° points toward its more reduced plane structure. As mentiond in the work of Sadasivuni et al., the peak near 25° increased while the peak near 11° decreased as the reduction temperature increased [45]. This trend suggests that raising the temperature within this range promotes an improved degree of reduction for rGO.

### 2.2. Properties

The semiconducting behavior dominated by thermally activated charge carriers and the metallic behavior dominated by charge carrier scattering are both observed in graphene, since its structure is determined by the graphene preparation method [25]. However, rGO shows quite consistent semiconducting behaviors due to the influence of functional groups on the band gap. A previous study showed that the band gap of rGO can be tuned by controlling the concentration of epoxide groups on the surface [34]. rGO has great conductivity and high thermal conductivity. It has been proved that the conductivity of GO can be significantly increased after chemical reduction [47], which is similar to graphene [48]. As for the electrical conductivity of rGO, it is controlled by the variable-range-hopping (VRH) of charge carriers between rGO layers and the simultaneous Arrhenius conduction within an rGO layer. The average distance between rGO layers (d-spacing) determines the thermally activated interlayer hopping of carriers. The conductivity of rGO significantly decreased after absorbing moisture from air due to the swelling effect of water on the d-spacing [34], which is one of the most important reasons to encapsulate rGO sensors. As for the temperature dependence, the electrical resistance of rGO decreases as temperature rises. This is because the probability of carrier scattering increases, which causes a decrease in the mobility of the charge carrier [15]. It has a sensitive thermal response to resistance changes and an excellent temperature coefficient of resistance, thus making it an ideal material for temperature sensing.

### 2.3. Assembly

**Figure 2:**
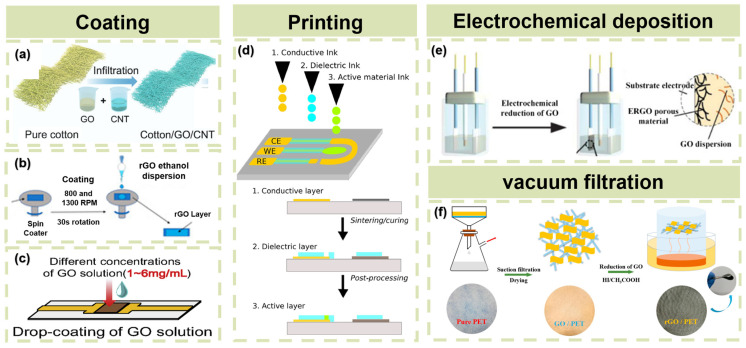
Assembly methods of rGO. (**a**) Schematic illustration of the preparation process of the Cotton/rGO/CNT composite. Reproduced with permission from Ref. [49]. Copyright 2022 Tsinghua University Press. (**b**) The spin coating method for sensing layer deposition. Reproduced with permission from Ref. [50]. Copyright 2022 by the authors. (**c**) Drop-coating of GO solution. Reproduced with permission from Ref. [51]. Copyright 2021 Elsevier Ltd. (**d**) Schematic representation of the fabrication of electrochemical sensors by inkjet printing. Reproduced with permission from Ref. [52]. Copyright 2017 Elsevier B.V. (**e**) Schematic illustration of electrochemical deposition method for preparing ERGO. Reproduced with permission from Ref. [53]. Copyright Royal Society of Chemistry. (**f**) Preparation process of rGO/PET and photos corresponding to pure PET, GO/PET, and rGO/PET. Reproduced with permission from Ref. [54]. Copyright 2018 Elsevier B.V.

Due to the diversity of substrates and reduction approaches, there are many reported assembly methods. Considering that rGO is mainly synthesized by reducing GO, spreading GO solution onto the substrate surface and then reducing it is a popular way to assemble rGO onto the substrate surface. The assembly methods are summarized in Figure 2, including coating, printing, electrochemical deposition, and infiltration.

#### 2.3.1. Coating

Coating methods mainly include spin coating, spray coating, dip coating, and drop coating. The spin coating (Figure 2b) method is carried out by first pouring solution on to substrate and then using a spin coater to spread the solution evenly over the surface [39]. A thin residual film is retained on the planner substrate after rotating due to the viscous force and surface tension [55]. Spray coating uses a spray gun to spray the solution onto the substrate [16,56]. A mask with a designed pattern can be applied to cover the substrate before spray coating to enable the patterning of sensors. Dip coating (Figure 2a) refers to immersing the substrate into a solution and then drying the film in air or a desiccator. If the substrate is absorbent, such as pure cotton, the solution will penetrate into the interior of the substrate, making the whole sample conductive instead of just staying on the surface layer. For drop coating (Figure 2c), the electrodes usually need to be prepared in advance, and then the solution dropped onto the reversed area between two electrodes. The conductive path will form after drying and reduction [51]. Coating is an assembly method suitable for depositing sensing layers in regular areas such as rectangles and circles. It remains limited in producing fine patterns.

#### 2.3.2. Printing

Printing methods mainly include screen printing and inkjet printing. Within the domain of printed circuits, screen printing stands as a well-developed technology, enjoying widespread popularity. A screen printer is used to squeegee the poured ink moving across the screen [57]. The printable inks used in screen printing usually contain metallic particles or wires, such as silver particle [22,58]. The main disadvantages of screen printing are a rough pattern surface due to the high viscosity of printable paste and a low resolution. Inkjet printing (Figure 2d) uses an inkjet nozzle head to deposit colloidal or solution in lines or droplets [59]. Inkjet printing is an entirely digital technique that uses inkjet printer to print ink on a predetermined track without any masks [60], which means less material consumption and thinner coating layers [52]. Compared with coating methods, printing shows more superiority in realizing specific patterns. Moreover, duplex printing has been reported to achieve an all-printed all-in-one integrated system [61].

#### 2.3.3. Electrochemical Deposition

Besides the three typical reduction methods mentioned above, in recent times, electrochemical reduction has emerged as a beneficial approach for achieving reduction. GO sheets near the working electrode are reduced and deposited onto the working electrode by immersing electrodes into a GO dispersion, which usually contains supporting electrolytes, and applying negative potential to the electrodes (Figure 2e) [53]. Electrochemical deposition can grow rGO film in situ and is able to produce 3D porous rGO hydrogel when the concentration of starting GO solution is sufficiently high [62]. Another important advantage of electrochemical deposition is that the thickness of rGO films can be easily controlled by changing the deposition time.

#### 2.3.4. Vacuum Filtration

Vacuum filtration is a popular method to prepare nanomaterial-based sensor structures. As shown in Figure 2f, vacuum filtration uses a vacuum pump to create a low-pressure environment on one side of the filter paper. The GO suspension is first dispersed with the help of ultrasonication or surfactant and then added to the top of substrate [59]. The pressure difference between the two sides encourages the solvent to pass through the filter paper, with the remaining GO sheets compactly and continuously attached to the substrate [54]. The rGO film is obtained after reduction, and its thickness is determined by the concentration of GO suspension initially used. The vacuum filtration method requires the substrate to be permeable to separate solvent and solute; thus, fibers and fabrics are ideal candidates. 

## 3. Temperature Sensing Mechanisms

The temperature dependence of rGO is determined by thermally activated charge carriers. As temperature increases, there is a higher probability of charge carriers moving from the valence band to the conduction band [15]. More specifically, the nonlinear behavior of resistance of the rGO film was observed in a study by Muchharla et al. [36]. Resistance increased sharply with decreasing temperature, below 180 K, while it increased slowly at higher temperatures. From 180 K to 400 K, which includes the normal body temperature sensing range (35 °C to 42 °C), the resistance (R) versus temperature (T) plot was included in this study using the equation displayed below:

(1) $$R=R_{0}exp(\frac{E_{g}}{k_{B}T})$$

where $E_{g}$ is the activation energy, $k_{B}$ is the Boltzmann constant, and $R_{0}$ is the resistance at infinite temperature. The ln(R) versus T^−1^ plot was best fit to a straight line in this temperature range, which indicates a band gap dominated by Arrhenius-like temperature dependence [63].

The temperature coefficient of resistance (TCR) is a vital parameter that was proposed to analyze the temperature sensitivity of materials. It can be defined by Equation (2) [64]:

(2) $$TCR=\frac{1}{R(T_{0})}\cdot \frac{R(T)-R(T_{0})}{T-T_{0}}$$

where R(T) is the resistance at temperature T, and R(T_0_) is the initial resistance of the tested sample at temperature T_0_. If the resistance decreases as temperature increases, then the calculated TCR value will be negative, which indicates the NTC (negative temperature coefficient) property. On the contrary, materials exhibit the PTC (positive temperature coefficient) characteristic when the TCR value is positive. 

## 4. rGO-Based Temperature Sensors

Due to the excellent properties mentioned above, rGO has been widely applied in wearable temperature sensors. Classified based on temperature-sensitive materials, the application of rGO in temperature sensors can be categorized into two types: pure rGO and composites of rGO with other materials. The addition of other materials usually aims to enhance the performance of rGO temperature sensors. The preparation, performance, and characteristics of these two types of temperature sensor are discussed in detail in this section. 

### 4.1. Pure rGO as the Active Material

**Figure 3:**
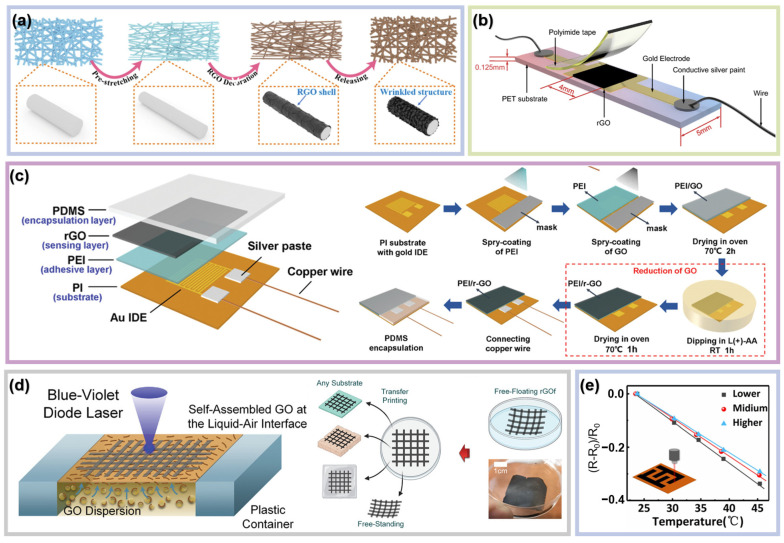
Temperature sensors based on pure rGO. (**a**) Schematic demonstration for the fabrication of the rGO shell assembly on a nanofiber surface. Reproduced with permission from Ref. [65]. Copyright 2021 Elsevier Inc. (**b**) Schematic of the temperature sensor, normalized resistance variation of the temperature sensor during heating and cooling processes, and normalized temperature response of the sensor at different bending angles. Reproduced with permission from Ref. [51]. Copyright 2021 Elsevier Ltd. (**c**) Schematic diagram of the temperature sensor structure and fabrication process of the skin-attachable temperature sensor. Reproduced with permission from Ref. [66]. Copyright 2019 WILEY-VCH Verlag GmbH & Co. KGaA, Weinheim. (**d**) Schematic showing the creation of rGOf through the direct laser writing reduction process of the self-assembled GO sheets at the liquid surface. The resultant rGOf is a free-floating fabric sheet, which can be either self-standing or transfer-printed onto any substrate, whether it is rigid or flexible. Reproduced with permission from Ref. [42]. Copyright 2022 WILEY-VCH GmbH. (**e**) Relative resistance variations as a function of temperature for different laser powers of laser writing for LIG-based interdigital electrode. Reproduced with permission from Ref. [29]. Copyright 2021 Elsevier B.V.

Wei et al. prepared an interface-engineered rGO temperature sensor assembly on a nanofiber surface [65]. To obtain the rGO and polyurethane (PU) structure, the pre-stretched PU nanofiber was immersed in the rGO/ethanol suspension. Ultrasonication treatment was applied to assemble rGO on the PU nanofiber surface. As shown in Figure 3a, the rGO sheets wrinkled after release due to the large rigidity mismatch between the rGO and PU nanofibers. The PU core provides high flexibility and stretchability, while the wrinkled rGO shell improves the material surface roughness and hence the hydrophobicity. This sensor achieved a TCR value of −0.36%/°C and a high linearity with R^2^ = 0.99, which can be used for temperature detection, health monitoring, and alarms. Chen et al. prepared a rGO/PET temperature sensor via laser reduction [51]. They first deposited gold film on PET substrate as electrodes by magnetron sputtering, then used a UV laser to reduce the GO coated on the center of the T-shaped electrode pair (Figure 3b). Polyimide tapes were used for packaging to prevent rGO from being oxidized. The sensor with the best performance showed a TCR value of −0.36%/°C, a high linearity of R^2^ = 0.999, and a fast response to thermal shock (0.196 s in response to iced water and 0.274 s in response to hot water). Moreover, it showed excellent repeatability and stability after the heating and cooling process and bending test, giving it great potential in electronic skins, human–machine interfaces, and other fields. Liu et al. reported a high-performance flexible temperature sensor based on rGO and a PEI bilayer [66]. The GO solution was uniformly deposited on the PEI layer by spray coating and reduced by dipping in the L(+)- ascorbic acid (L(+)-AA) solution (Figure 3c). Chemical bonding between rGO and PEI was formed through an amidation reaction, whereby PEI penetrated into graphene-interlaced sheets though the redissolution phenomenon, which provided the opportunity for carriers on the rGO sheets to break through the barrier and transfer to the adjacent rGO sheets via the tunneling effect. This special bilayer structure increases the sensitivity of the sensor, which realized a −1.30%/°C TCR value with R^2^ = 0.999 and a 0.1 °C resolution from 25 °C to 45 °C. Due to its excellent sensitivity, reliability, and flexibility, this temperature sensor is considered to have potential in the fields of disease diagnosis and healthcare. Liu et al. reported an rGO fabric prepared by direct laser writing at the interface between liquid and air that can be used for temperature sensing [42]. As shown in Figure 3d, the rGO patterns applied to the liquid surface can be transferred onto any other needed substrate without considering laser ablation, since water has a high heat capacity to protect sensors from thermal degradation. This device is not only a temperature sensor but also a heater. The temperature of the rGO fiber adhered on an insulative glass substrate can reach the maximum in less than 0.5 s by applying DC voltage to both sides of the electrical conductor. It can be used to provide heat for the human body when facing threats from a low-temperature environment or diseases that cause temperature loss. Han et al. reported a temperature sensor based on rGO and laser-induced graphene (LIG) [29]. The LIG interdigital electrode was obtained by direct laser writing and the rGO sensing film was prepared by drop coating and thermal reduction. The temperature sensor exhibited a high sensitivity of −1.30%/°C over a range from 25 °C to 45 °C with a resolution of 0.2 °C. In addition, our investigations show that the temperature sensing performance is affected by thermal reduction temperature, laser power, and the finger spacing of the interdigital electrodes. As shown in Figure 3e, the sensitivity of rGO/LIG temperature sensors decreased as the direct laser writing power increased. The rise in laser power corresponds to an increase in defects within the LIG under a certain range, which influences the charge carrier transport [67,68].

### 4.2. rGO Composite as the Active Material

**Figure 4:**
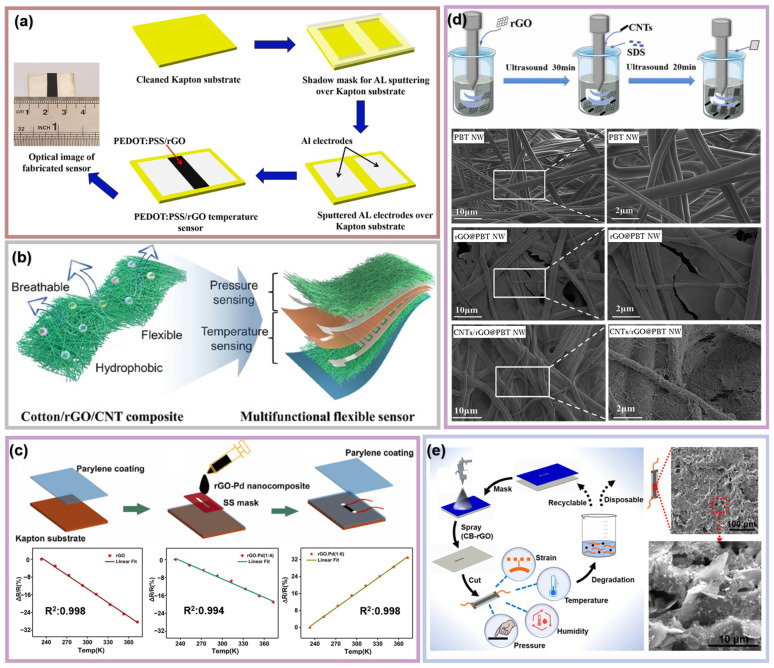
Temperature sensors based on rGO mixed with other materials. (**a**) The schematic of the fabrication process for PEDOT:PSS/rGO temperature sensor. Reproduced with permission from Ref. [69]. Copyright 2022 the author(s), under exclusive license to Spring Science Business Media, LLC, part of Spring Nature. (**b**) Schematic illustration of the multifunctional flexible sensor. Reproduced with permission from Ref. [49]. Copyright 2022 Tsinghua University Press. (**c**) rGO-Pd temperature sensor fabrication and the proposed sensor’s temperature performance. Reproduced with permission from Ref. [20]. Copyright 2021 Elsevier B.V. (**d**) Schema of the fabrication process for rGO/CNTs@PBT MB temperature sensor and the SEM images. Reproduced with permission from Ref. [70]. Copyright 2022 Elsevier B.V. (**e**) Schematic illustration of the preparation process and the SEM images of the sensitive layer (sprayed composite of CB and graphene) on the paper substrate. Reproduced with permission from Ref. [16]. Copyright 2019 American Chemical Society.

Seifi et al. developed a high-sensitivity wearable temperature sensor based on PEDOT:PSS/rGO on a flexible Kapton substrate by first sputtering aluminum electrodes and then drop casting PEDOT:PSS/rGO ink onto the substrate (Figure 4a) [69]. Among conductive organic thermoelectric materials, PEDOT:PSS stands out with the highest efficiency, attributed to its composition, comprising PSS with a negative charge and PEDOT with a positive charge. PEDOT:PSS is also a popular temperature sensing material with NTC properties. This study concluded that the environment stability of temperature sensors can be improved by adding rGO to the PEDOT:PSS; to be specific, the difference between heating and cooling curves is smaller when using the composite of rGO and PEDOT:PSS than using PEDOT:PSS only. This sensor exhibited a −3.36%/°C TCR value with R^2^ = 0.9904 at the temperature range of 30–45 °C. The response and recovery times are 2 s and 7 s, respectively, which is suitable for real-time body temperature monitoring. Yin et al. reported a waterproof and breathable cotton/rGO/CNT composite multifunctional flexible sensor [49]. As shown in Figure 4b, the sensor contains six layers in total, with the PI substrate film located in the middle to separate the temperature and pressure sensing parts. As a flexible and skin-friendly substrate, pure cotton was immersed into GO dispersion and then CNT solution to absorb these two sensing materials. This sensor showed a great linear response ranging from 28 to 40 °C. Different from normal rGO temperature sensors, the resistance of this device interestingly increased as temperature rose (PTC property). This is because the cotton/rGO/CNT composite expanded as temperature increased, resulting in a decreased contact area between the fibers coated with rGO and CNT. This proves that the performance of temperature sensors based on composites is jointly determined by each component. Nuthalapati et al. developed a highly sensitive strain and temperature sensor based on graphene palladium (Pd) nanocomposite on Kapton substrate [20]. The rGO-Pd nanomaterial was prepared by incorporating rGO with Pd nanoparticles at a predefined ratio using an organic solvent like N-methyl-2 pyrrolidone. Then, the sensing film was prepared by screen printing, as shown in Figure 4c. By analyzing the experimental results, it can be concluded that the ratio of Pd and rGO strongly influenced the temperature sensing performance of the composite sensor. The TCR values of the composite temperature sensors with rGO-Pd ratios of 1:0, 1:2, and 1:4 are negative, while those with rGO-Pd ratios of 1:6 and 1:8 are positive, implementing flipping from NTC to PTC. The absolute value of the TCR decreased as the Pd percentage increased, which means higher temperature sensitivity. Wang et al. developed a flexible temperature sensor based on rGO and CNTs with PBT nano melt-blown nonwoven fabric (PBT NW) by immersing the PBT NW into the rGO and CNT dispersed conductive suspension and performing ultrasonication for 20 min [70]. The homogeneous conductive suspension was prepared by adding a certain amount of CNTs into rGO suspension and then stirring for 30 min. The sensitivity of temperature sensors fabricated by single graphene is often inadequate [71] since the conductive pathway composed of two-dimensional conductive graphene is easily destroyed during the process of heating and cooling [72], which leads to a poor temperature sensing performance. By introducing one-dimensional linear conductive material CNTs and combining CNTs with rGO, a robust conductive network structure was formed, with the one-dimensional CNTs bridging the conductive path of the graphene-based temperature sensor (Figure 4d). Moreover, the intensity of the diffraction peaks of rGO and CNTs shown in the XRD pattern became weaker after combining, suggesting that the strong interaction among rGO, CNTs, and PBT NW results in the alterations in crystallinity level [73]. The special conductive network structure led to high temperature sensitivity (−0.737%/°C) and high linearity (R^2^ = 0.98), exhibiting great performance in detecting body temperature and human respiration [70]. Liu et al. reported a flexible sensor based on rGO and carbon black (CB) hierarchical composite on paper substrate that could detect strain, humidity, temperature, and pressure [16]. A plastic mask was covered on the paper substrate before the dispersion of CB and rGO mixture was sprayed on the paper to form a designed pattern (Figure 4e). Through iterative rounds of spraying and drying, the CB particles were compacted with cracks and absorbed on the surface of rGO, while the sprayed rGO yielded even layers of sheets which were then overlapped together. This hierarchical structure resulted in a high TCR of −0.6%/°C from 20 °C to 60 °C. Zhu et al. prepared a highly sensitive flexible temperature sensor based on the composite of single-wall carbon nanotubes (SWCNTs), PEDOT:PSS, and rGO on PI substrate [74]. The PEDOT:PSS solution was added into the mixture solution of functionalized SWCNTs and rGO. After ultrasonication, the dispersed solution was vacuum filtered to obtain a solid sample. This temperature sensor provided a high TCR value of −0.33%/°C with R^2^ = 0.99 between 30 °C and 45 °C and displayed a fast response and recovery time (5.9 s and 6.2 s, respectively) between 25 °C and 38 °C, which was used for temperature sensing, exhaling, and the detection of the spatial distribution in response to external stimuli. 

## 5. Applications Based on rGO Temperature Sensors

**Figure 5:**
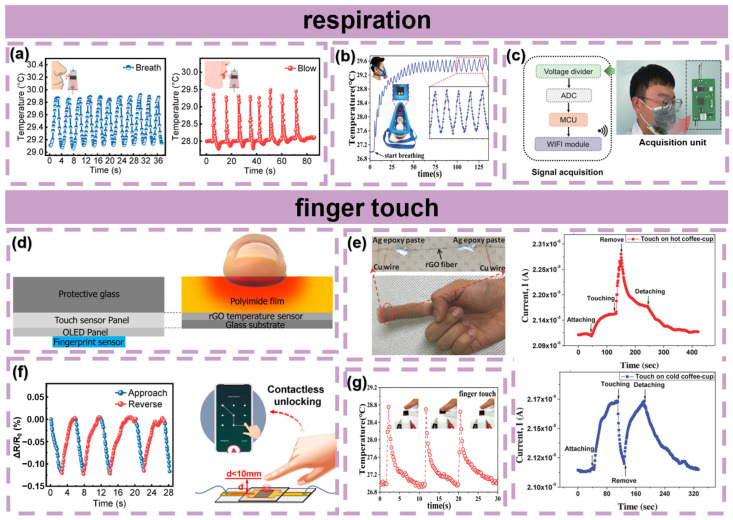
(**a**) Sensor application test about breathing rate monitoring and human blowing detection. Reproduced with permission from Ref. [51]. Copyright 2021 Elsevier Ltd. (**b**) Breathing rate monitoring inside a gas mask. Reproduced with permission from Ref. [66]. Copyright 2019 WILEY-VCH Verlag GmbH & Co. KGaA, Weinheim. (**c**) A demonstration system realizing the non-contact, smart, and real-time monitoring of human respiration signals. Reproduced with permission from Ref. [49]. Copyright 2022 Tsinghua University Press. (**d**) rGO temperature sensor with buffer layer of polyimide film. Reproduced with permission from Ref. [75]. Copyright 2021 Elsevier B.V. (**e**) Temperature response of the device worn on a human finger and the response current of the device to the temperature of skin touching a hot coffee cup and a cold coffee cup. Reproduced with permission from Ref. [4]. Copyright 2018 WILEY-VCH Verlag GmbH & Co. KGaA, Weinheim. (**f**) Proximity detection experiment. (A color version of this figure can be viewed online. Reproduced with permission from Ref. [51]. Copyright 2021 Elsevier Ltd. (**g**) Finger touch detection. Reproduced with permission from Ref. [66]. Copyright 2019 WILEY-VCH Verlag GmbH & Co. KGaA, Weinheim.

**Figure 6:**
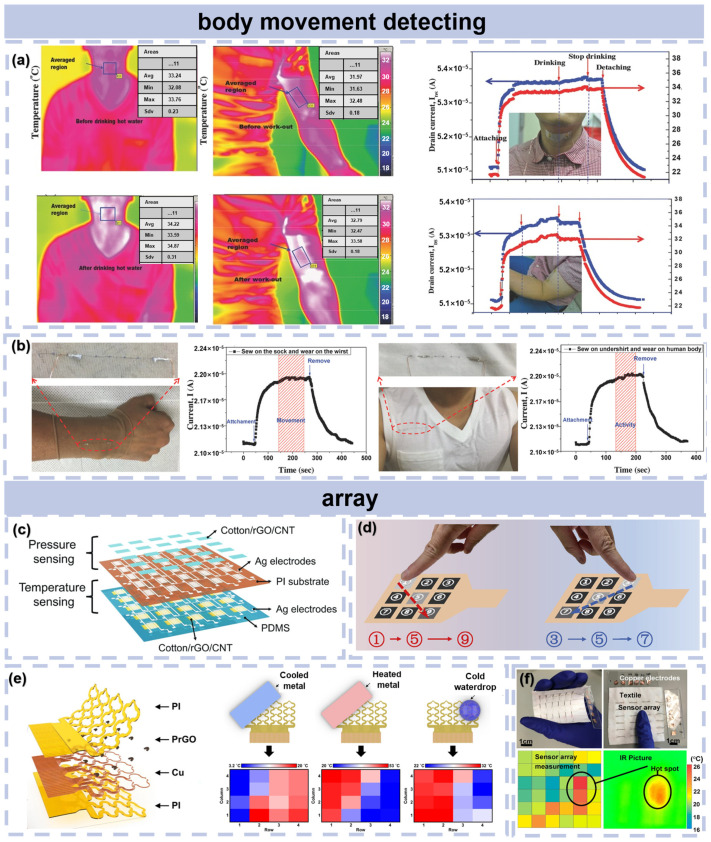
(**a**) Transparent and stretchable integrated platform of temperature and strain sensors responding simultaneously to the temperature of human skin and to muscle movement. Reproduced with permission from Ref. [79]. Copyright 2015 WILEY-VCH Verlag GmbH & Co. KGaA, Weinheim. (**b**) Monitoring the temperature of the human body during movement and activity. Reproduced with permission from Ref. [4]. Copyright 2018 WILEY-VCH Verlag GmbH & Co. KGaA, Weinheim. (**c**) Schematic illustration of the sensor array of multifunctional flexible sensors. Reproduced with permission from Ref. [49]. Copyright 2022 Tsinghua University Press. (**d**) Code lock application of the nine-channel flexible temperature sensor. Reproduced with permission from Ref. [51]. Copyright 2021 Elsevier Ltd. (**e**) Schematic illustrations of the PrGO temperature sensor array mounted on a human wrist and response of the sensor to the metal and water drop with different temperatures placed on the sensor array. Reproduced with permission from Ref. [19]. Copyright 2023 American Chemical Society. (**f**) The thumb touch test of the textile-infused array of 6 × 6 temperature sensors. Reproduced with permission from Ref. [80]. Copyright 2018 Elsevier B.V.

The application of rGO in temperature sensors has developed rapidly. Body temperature sensing is the most basic function of rGO temperature sensors. Based on the same temperature sensing mechanism, they are also used to detect respiration, body movement, and finger touching. When it was realized that obtaining physiological signals from a single point is not enough, sensing arrays were developed to achieve a regional signal distribution. All of these applications are classified and shown in Figure 5 and Figure 6. 

### 5.1. Respiration

Respiration is also an important vital signal in health monitoring. Respiration data can be obtained by sensing the temperature of exhaled gas. As shown in Figure 5a, when the temperature sensor was positioned in front of the nostrils of a volunteer, the regular temperature changes reflected that the respiration rate was 18 times per minute. The air temperature changes could also be recorded when the top of sensor was blown. To obtain more accurate respiration data, another testing method is fixing the temperature sensor on the inner side of a gas mask (Figure 5b). The regular ups and downs in the tested temperature in the relatively closed space accurately reveal the breathing rate. Similarly, the relatively closed space can also be provided by a normal mask, where researchers have reported putting sensors inside to monitoring people’s breathing in the context of coronavirus diseases. As for signal acquisition, Yin et al. used a system including an analog-to-digital converter (ADC) circuit, a micro-controller unit (MCU), and a WIFI transmission module to display the detected real-time respiration results on a terminal screen (Figure 5c) [49]. 

### 5.2. Finger Touch

Nimble fingers are important tools to interact with the outside world, and measuring the temperature of fingers can help to analyze the position and movement of fingers. As shown in Figure 5g, the temperature of a sensor rapidly rose when it was touched by a finger and slowly decreased after it was removed, which is the most basic example of finger touch sensing. Figure 5d exhibits another example that used a semi-transparent rGO temperature sensor for real-time fingerprint sensing on the display screen of a smartphone. The temperature rose where the fingers touched, resulting in changes in the rGO film’s resistance. Verifying the temperature of fingers can prevent potential falsification during fingerprint authentication. Figure 5e demonstrates the response current of the rGO temperature sensor worn on a finger when touching a hot and a cold coffee cup. The current change points revealed the changes in finger states, including attaching, touching, removing, and detaching. Besides direct touching, rGO temperature sensors are also widely applied in the field of contactless sensing. For example, touchless elevator buttons have been put into use to reduce disease risks from direct contact. A similar application is shown in Figure 5f: the resistance decreased sharply when a finger approached the sensor and recovered rapidly to the original value when the fingertip was removed. Different from the common proximity sensors based on the use of capacitive [76], electromagnetic [77], and inductive sensors [78], proximity sensors based on temperature differences operate without being constrained by the material of the object and exhibit a remarkable level of stability [51]. 

### 5.3. Body Movement Detecting

Figure 6a shows an application in body-attachable wearable electronics based on a transparent and stretchable rGO temperature sensor. As shown, the temperature of the neck skin surface increased from 33.2 °C to 34.1 °C during the drinking of hot water, which was attributed to the body’s heat release mechanism for temperature control, and the temperature of the front arm increased from 31.7 °C to 32.6 °C during dumbbell exercises. This implies that temperature sensors can achieve the continuous tracking of human skin temperature and muscle motion in real time during various human activities. The rGO temperature sensors can also combined with textiles to directly and comfortably make contact with the skin. Figure 6b shows the response current of rGO temperature sensors worn on the wrist while the hand moved up and down. It can be observed that these currents remained nearly constant during wrist movement and activities, which indicated great advantages in healthcare and biomedical monitoring.

### 5.4. Arrays

Arrays have become a trend in flexible temperature sensing. Figure 6c demonstrates an example of a sensing array (5 × 5 pixels), which was designed to realize the spatial arrangement of pressure and temperature information from the contacted object, achieved by repeating rectangle sensing pads [46]. Figure 6e exhibits another array pattern consisting of 16 channels that worked independently. This PrGO temperature sensing array monitored the distribution of thermal mapping, which is much more reliable than a single spot. Figure 6e illustrates the heat distribution patterns across the sensing array in response to localized applications of cooled metal, heated metal, and cold-water droplets. The sensing array is capable of distinguishing the variations in temperature across different regions of human skin, which is potential in biomedical applications. Figure 6f demonstrates a textile-infused temperature sensing array that could highlight the area touched by finger. It was found that the spatial resolution of this textile-infused sensor array depends on the diameters of rGO-coated filaments and conductive threads. Figure 6d shows a 3 × 3 flexible temperature sensor array that was designed for the contactless unlocking of a code lock as a human–machine interface. The back-end system identified the normalized resistance change in each individual channel; the order of resistance changed when the temperature-sensitive units matched the password.

## 6. Conclusions

Given that body temperature serves as a reflection of various abnormal bodily conditions, temperature sensing and monitoring assume a pivotal role in healthcare, disease prevention, and prosthetic skin applications. This paper delves deep into the principle, design characteristics, and application fields of wearable temperature sensors based on reduced graphene oxide (rGO), a temperature-sensitive material. The aim is to comprehensively understand the immense potential and wide-ranging application prospects of this novel material in wearable temperature sensors. rGO can function as the sole temperature-sensitive material in the sensor, or it can be combined with other temperature-sensitive materials such as Ag particles, carbon nanotubes, graphene, and PEDOT:PSS to enhance sensor performance. Beyond conventional body temperature sensing, wearable temperature sensors crafted from rGO films can extend their utility to respiratory rate monitoring, finger touch detection, and body movement perception. As application requirements grow, the development trend in flexible sensors is leaning towards temperature sensor arrays to yield more precise data and spatial signal distributions. 

Wearable temperature sensors founded on reduced graphene oxide hold substantial potential within the healthcare domain, but they are accompanied by a series of challenges. Foremost among these is ensuring the accuracy and stability of flexible temperature sensors, particularly their consistent performance across diverse environmental conditions in terms of temperature and humidity. Moreover, designing these temperature sensors should also carefully address users’ comfort prerequisites. Despite these challenges, the future of wearable temperature sensors appears promising. Ongoing advancements in sensing technology, data analysis algorithms, and human–computer interaction are poised to further elevate the role of wearable body temperature sensors across various domains. This progress presents a plethora of possibilities for enhancing individuals’ health management and quality of life. 

## Data Availability

Data are available in a publicly accessible repository.
